# FISim: A new similarity measure between transcription factor binding sites based on the fuzzy integral

**DOI:** 10.1186/1471-2105-10-224

**Published:** 2009-07-20

**Authors:** Fernando Garcia, Francisco J Lopez, Carlos Cano, Armando Blanco

**Affiliations:** 1 Department of Computer Science and Artificial Intelligence, University of Granada, Granada 18071, Spain

## Abstract

**Background:**

Regulatory motifs describe sets of related transcription factor binding sites (TFBSs) and can be represented as position frequency matrices (PFMs). De novo identification of TFBSs is a crucial problem in computational biology which includes the issue of comparing putative motifs with one another and with motifs that are already known. The relative importance of each nucleotide within a given position in the PFMs should be considered in order to compute PFM similarities. Furthermore, biological data are inherently noisy and imprecise. Fuzzy set theory is particularly suitable for modeling imprecise data, whereas fuzzy integrals are highly appropriate for representing the interaction among different information sources.

**Results:**

We propose FISim, a new similarity measure between PFMs, based on the fuzzy integral of the distance of the nucleotides with respect to the information content of the positions. Unlike existing methods, FISim is designed to consider the higher contribution of better conserved positions to the binding affinity. FISim provides excellent results when dealing with sets of randomly generated motifs, and outperforms the remaining methods when handling real datasets of related motifs. Furthermore, we propose a new cluster methodology based on kernel theory together with FISim to obtain groups of related motifs potentially bound by the same TFs, providing more robust results than existing approaches.

**Conclusion:**

FISim corrects a design flaw of the most popular methods, whose measures favour similarity of low information content positions. We use our measure to successfully identify motifs that describe binding sites for the same TF and to solve real-life problems. In this study the reliability of fuzzy technology for motif comparison tasks is proven.

## Background

One of the main goals in computational biology is to understand how expression of genes is controlled, and to unravel gene regulatory networks. Cells control the abundance and activity of proteins by means of diverse factors in which transcription regulation plays a central role. Multiple events are involved in the initiation of transcription of a gene. One of the most important ones is the binding of several proteins, called transcription factors (TFs), to DNA near the gene, called transcription factor binding sites (TFBSs). TFBSs are usually located close to the transcription start site (TSS) of the gene and upstream from it. Additionally, in some cases TFBSs can be found downstream the TSS or, in rare instances, even within exons [1]. These interactions between DNA and proteins play a crucial role in controlling the expression of the genes by activating or inhibiting the transcriptional machinery.

The identification of binding sites bound by transcription factors is therefore a key problem in predicting transcription regulation. Sometimes a given TF can bind to only one TFBS, but usually the same TF can bind to different DNA sequences and its binding preferences are represented by means *regulatory motifs*. The recognition of de novo TFBSs usually includes the issue of comparing putative motifs with one another and with motifs that are already known. Many studies discuss the advantages of different regulatory motifs representations [2]. Regulatory motifs are usually presented as matrices representing the binding affinity of the TFs, derived from a multiple alignment of confirmed binding sites for a given transcription factor. Most existing approaches for computing motif similarity represent the motifs by means of position frequency matrices (PFMs) that record the position-dependent frequency of each nucleotide, or position weights matrices (PWMs) of score values that give a weighted match to any given substring of fixed length. With the emergence of high-throughput technologies (e.g. ChIP-chip assays, DNA microarrays, etc.) numerous algorithms for finding motifs have appeared (for a review see [3]). These algorithms usually filter their outputs in order to improve their significance, e.g. merging similar motifs. However, the outcome of these tools, particularly when dealing with large datasets, is usually presented as a large list of motifs that require further post-processing in order to make it meaningful. Methods for comparing motifs are usually applied to give biological significance to the outputs of these programs. This is usually done by comparing the putative motifs provided by these algorithms against known motifs reported in motif databases such as JASPAR or TRANSFAC [4,5]. Unveiling these relationships might be crucial for the design of appropriate biological experiments.

The existing motif discovery algorithms make use of different strategies to overcome drawbacks of other approaches, usually implying new or different limitations. One common approach involves using several of these algorithms and compounding their outputs [6]. In this case, motifs found by different algorithms can either correspond to the same TFBSs or to different ones, making the compounded result very noisy and imprecise. This suggests a need for comparison methods for finding similar motifs to be either removed or merged into a new motif.

The most common strategy relies on the assumption that the columns of the matrices are probability distributions. Thus, most measures between motifs are based on statistical techniques that test whether the different columns belong to the same distribution. Pietrokovski [7] used a straightforward algorithm based on the Pearson correlation coefficient (PCC). Wang and Stormo [8] proposed the average log-likelihood ratio (ALLR) to compare between motif columns. Schones et al. [9] made the comparison by means of a Pearson *χ*^2 ^test (PCST). They also proposed the Fisher-Irwin exact test (FIET) which provided poorer results. In addition, the Kullback-Leibler divergence (KLD) was used to compare motifs [10]. Rather than comparing distributions, Choi et al. [11] used the euclidean distance (ED) between columns, obtaining promising results. In addition, Gupta et al. [12] developed an algorithm (Tomtom) that allows any column-to-column measure. They compute *p*-values of the match scores for the columns of the query motif aligned with a given target motif. They obtained best results when using euclidean distance. More recently, Pape et al. [13] introduced the concept of a *natural *measure between motifs. They proposed that two motifs should be considered to be similar if they yield a high number of overlapping hits on a random sequence. They considered the number of hits as a random variable and described a method based on covariance to measure the correlation between the random variables of two PFMs.

In recent years, it has been seen that the inherent uncertainty and noise that characterize biological data cannot always be modeled sufficiently well by probabilistic approaches and that, consequently, alternative models for gathering this uncertainty may be required. Furthermore, in the context of motif comparisons, the utilization of PFMs as a representation of the binding preferences of the TFs inherently includes imprecision. In addition to the usual missing values and noisy data associated with biological data, there exist some *hidden *factors apart from the DNA sequence itself that affect the binding preferences of TFs, e.g. cooperative binding and chromatin structure [14]. Moreover, an arbitrary threshold must usually be chosen in the construction of a PFM itself.

Although existing methods have been shown to work well, there is still room for improvement. Several properties are desirable for a motif similarity measure:

• Greater importance should be given to the similarity of high information content positions of the motifs than to the similarity of low information content positions.

• Methods should be designed to deal with the inherent uncertainty associated with motif comparison tasks.

• The use of parameters should be minimized.

Existing methods fail to follow one or more of these considerations. In general their approaches are not designed to deal with imprecise scenarios. In addition, these methods are not designed to consider the higher contribution of better conserved positions to the binding affinity. Some methods intrinsically tend to give greater importance to better conserved positions (e.g. ED). However, this can be improved. There is therefore a need for similarity measures for motifs that deal with these kinds of problems. In this paper we present FISim (Fuzzy Integral Similarity), a novel similarity measure for comparing two motifs with one another based on the fuzzy integral with respect to a fuzzy measure.

Zadeh [15] proposed fuzzy set theory to mathematically model the imprecision inherent to some concepts. Briefly, fuzzy set theory allows an object to partially belong to a set with a membership degree between 0 and 1. Classical set theory is a special case of its fuzzy counterpart in which membership and certainty degrees are restricted to either 0 or 1. Fuzzy theory is especially suitable for dealing with imprecise, noisy and uncertain environments. It has been successfully applied to many different areas, including control, pattern recognition, and data mining, e.g. classification and clustering [16]. In recent years, some works have appeared that integrate fuzzy solutions to solve biological problems like microarray analysis, protein location, etc., showing promising results [1,17].

One of the most popular tools for information aggregation is the weighted average method. It is simple, intuitive and easy to implement. This method assumes that the different information sources are non-interactive/independent and, hence, their weighted effects are viewed as additive. Due to some inherent interaction/inter-dependencies among diverse information sources, the weighted average method does not work well in many real problems. In our case, the affinity of a TF to a specific TFBS is typically correlated with how well the site matches the consensus sequence of the corresponding motif. However not all mismatches at a given position have the same effect and some interactions between positions have been observed [18]. In this paper we propose the use of the fuzzy integral to formally incorporate the different degrees of importance of the positions according to their infomation content level. Fuzzy integrals are a type of non-linear function dependent on fuzzy measures, and have been shown to be very useful for multiple information source fusion [19,20]. The combination of multiple information sources is very valuable with regard to overcoming the inherent ambiguities present in single information sources. Fuzzy integrals are capable of representing the interaction among the information sources (e.g. motif columns) and of combining them to make the result more significant than just the sum of the individual comparisons, enabling the individual importance of each source to be considered in the final result (e.g. information content level).

FISim is intended to meet these requirements. First, greater importance is given to the similarity of higher information content positions via the fuzzy integral, according with the biological binding properties of TFs to TFBSs (more details in Methods section). Second, it is based on fuzzy technology and is intended to deal with the intrinsic uncertainty involved in motif comparison tasks. Third, FISim does not require the user to have any previous knowledge, as it does not need any user-provided parameter. In what follows, we use the term *conservation *to refer to the information content level of the motif positions.

As explained above, one of the main applications of a similarity measure for motifs is as the basis for clustering procedures for grouping related motifs together. Previous studies either make use of hierarchical clustering methods [21] or define modifications of the PAM algorithm to obtain the grouping [13]. In this research, we present a novel clustering methodology termed kcmeans (kernel c-means) based on kernel methods and the c-means algorithm combined with our FISim measure. Familial Binding Profiles (FBPs) are generalized binding profiles that can be used as the representatives of their respective group of motifs [22]. In our proposed methodology, we automatically compute FBPs for the clusters from a multiple alignment of the motifs within each cluster. We use kcmeans to cluster motifs obtained from the JASPAR database [4], and we compare our results with those from existing approaches.

## Results

### Distinguishing randomized motifs

#### Random motifs

We tested the performance of FISim in measuring the differences between sets of random motifs. We considered 20 randomly generated *seed *motifs of a fixed length of 6 nucleotides. Following the JASPAR motif properties, the information content was uniformly ranged from 1.5 to 10.5 (for some JASPAR motif statistics see additional file 1: "JASPAR motif statistics"). For each one of the 20 *seed *motifs, a true dataset was generated containing 10000 motifs. In order to match with the properties of real motifs [23], each motif in the true datasets was obtained as follows:

A *random *motif of a random length between 6 and 14 was generated. The information content of this *random *motif is controlled to be low in order to create a non-conserved flanking region for the motif. The corresponding *seed *motif was sampled from a Dirichlet distribution with a random sample size between 25 and 35 [9], which generated a *sample *motif of length 6. Finally, starting in a random position, the columns in the *random *motif are replaced by the *sample *motif.

**Figure 1:**
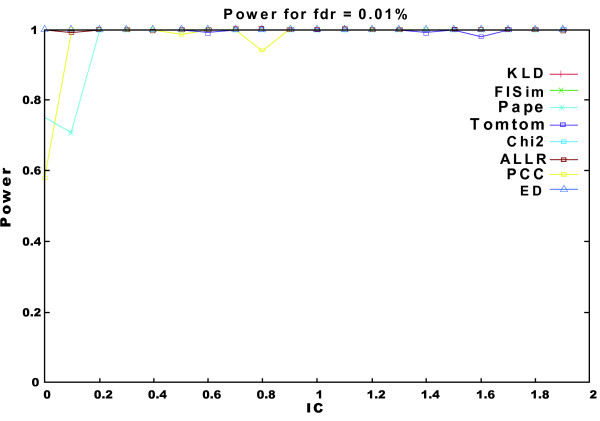
**Random Motifs**. Power of the methods to recognize random PFMs generated by the same distribution.

Similarly, a false dataset was generated. The process is the same as for the true datasets but we omitted the insertion of samples from *seed *motifs and the information content is not controlled. Figure 1 shows the power (selectivity) of the methods in recognizing motifs generated from the *seed *motifs when the FDR is 0.01. FISim shows a very good performance in a random dataset.

### Distinguishing conserved and non-conserved motifs

#### Case study

**Figure 2:**
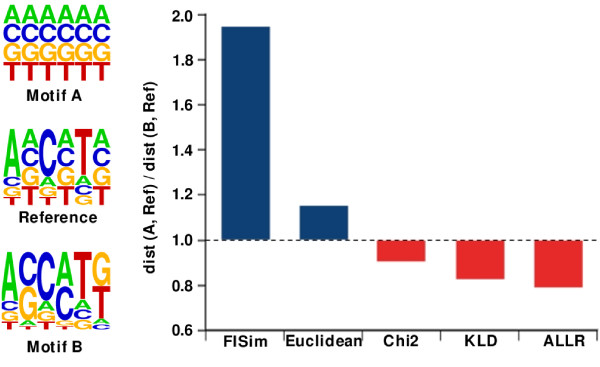
**Case study**. Ratio of distances. In order to facilitate the visual comparison of the non-conserved positions, fraction-based logos are used. We do not show results for the measures proposed by Pape et al. [13] nor Gupta et al. [12] since they need a background dataset to work properly.

We wanted to demonstrate the ability of the measures in discriminating the importance of non-conserved positions and well-conserved positions. In Figure 2 we show three motifs. We used the middle one as a *reference*. It has well-conserved positions in the odd locations (permutations of the column vector [10, 2, 2, 2]), and non-conserved positions in the even locations (from column vector [4, 4, 4, 4]). This *reference *motif was compared with the other two motifs to check how each measure performs:

• Motif *A *is composed of non-conserved columns. It therefore matches perfectly with the even positions of the *reference *motif. However, the similarity between odd positions (well-conserved) is expected to be low.

• Motif *B *is made up of two kind of columns: *a) *well-conserved positions in the odd locations that match perfectly with the corresponding positions of the *reference *motif, and *b) *medium-conserved positions (derived from permutations of the vector [7, 7, 1, 1]) in the odd locations that differ from the odd positions of the *reference *motif.

Note that both motifs *A *and *B *perfectly match half of the positions of the *reference *motif, while they differ in the other half of the positions. These differences are controlled for balance, in the sense that the *raw distance *of the different positions is the same, e.g. *raw distance *between [10, 2, 2, 2] and [4, 4, 4, 4] (*reference *motif and motif *A *differences) equals to the *raw distance *between [4, 4, 4, 4] and [7, 7, 1, 1] (*reference *motif and motif *B *differences). We call *raw distance *to the sum of the absolute value of the four differences between the counts of the nucleotides of the two columns.

We then considered two cases for each of the measures: *case 1 *: distance between motif A and the *reference *motif, and *case 2 *: distance between motif B and the *reference *motif. As has been explained above, it would be desirable that the distance for *case 2 *be lower than the distance *case 1*, as, unlike motif *A*, motif *B *and the *reference *motif share the similarities in the most conserved positions of the motifs. In Figure 2 we show the ratio of the distances for *case 1 *against *case 2*. Results for the measures proposed by Gupta et al. and Pape et al. [12,13] are not shown since they require a background dataset to function correctly. Three of the measures (*χ*^2^, KLD and ALLR) failed to capture the expected differences, and provided a lower distance for *case 1*. On the other hand, our measure obtained a more realistic distance between the motifs, providing a much lower distance for *case 2*,

#### Related motifs

**Figure 3:**
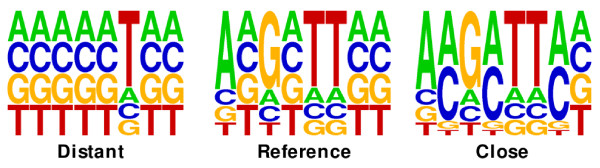
**Related motifs**. Three dissimilar positions are observed between the *reference *motif and both *close *and *distant *motif. Again, fraction-based logos are used to ease the visual comparison of the non-conserved positions.

**Figure 4:**
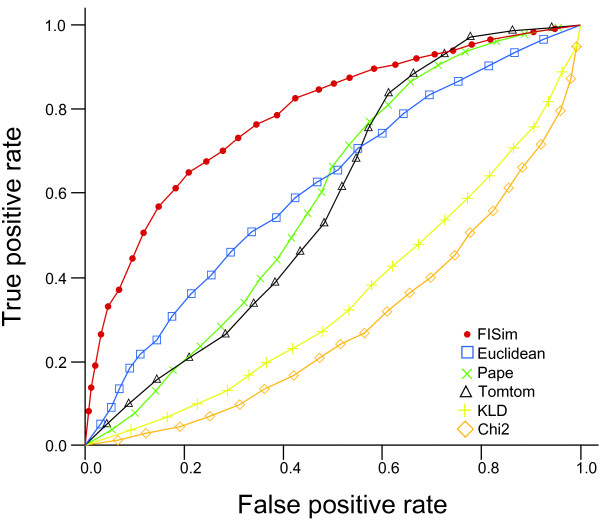
**ROC curves**. ROC curves for the case of three different columns. FISim provides a more consistent classification than the rest of the methods.

We extended the last experiment to check the performance of the methods in datasets of related motifs. We generated a *reference *motif of length 8 comprising four well-conserved positions and four non-conserved positions used as a *reference *(see previous section for more details). We then obtained a pair of *seed *motifs comprising one *close *motif and one *distant *motif with respect to the *reference *one. Each of these motifs present three positions dissimilar to the *reference *motif. The *close *motif present the dissimilarities in the non-conserved positions, while the *distant *motif present the dissimilarities in the conserved positions (Figure 3). We generated a true dataset for the *close *motif and a true dataset for the *distant *motif following the procedure of above experiments. For each motif in the datasets we computed its distance to the *reference motif*. We determined a correct classification when a smaller distance is assigned to the *close *motif, and determined an incorrect classification otherwise. We arranged the motifs according to their distances, and from this arranged set of motifs we computed an ROC (Receiver Operating Characteristic) curve [24]. ROC curves plot the percentage of correct classifications as a function of incorrect classifications. In Figure 4 we show the ROC curves obtained from the different approaches. It can be seen that our FISim method proposed outperforms the other methods. Similar results are obtained when varying the number of dissimilar positions of the seed motifs. The area under the curves (AUC) scores and the logos for the motifs can be found in the additional file 2: "Related motifs experiment".

### Clustering real data

In order to check the performance of kcmeans in separating related motifs, we used the freely accessible JASPAR [4] database for our experiments. JASPAR contains 71 nonzinc-finger motifs divided into 11 classes according to the structural properties of the transcription factors. The distribution of the families of the JASPAR motifs can be found in Table 1. For each motif we computed the core region, following the suggestions of Schones et al. [9]. In order to obtain a symmetric matrix, comparisons between two motifs were made by averaging the similarity between the core region of the first motif and the second motif, and the similarity between the first motif and the core region of the second motif. Once we obtained the similarity matrix, we applied the *kcmeans *clustering method as described in the Methods section. For each cluster, the FBP is automatically obtained from a multiple alignment of its corresponding motifs.

**Table 1:** JASPAR family distribution

Family	Number of motifs	Family	Number of motifs
ETS	7	TRP	5
FORKHEAD	8	HMG	6
bHLH	10	HOMEO	8
bZIP EBP	4	NUCLEAR	8
MADS	5	bZIP CREB	4
REL	6		

Summary of the JASPAR classification. There exist 71 motifs divided into 11 families.

To obtain the optimal number of clusters (*k*) we used the Silhouette coefficient [25]. The optimal clustering of the 11 motifs classes was found for *k *= 15. The 15 clusters and the logos of the motifs within each cluster can be found in additional file 3: "JASPAR clustering". To ensure the quality of the clustering, we compared our results with those provided by Pape et al. [13].

Two identical clusters are obtained: NUCLEAR and bZIP CREB. The same MADS and HOMEO groups are provided but we yielded a MADS motif (*MEF2A*) within the HOMEO group. MADSs motifs present the consensus CCA*A, while HOMEO motifs present the consensus ATTA. *MEF2A *motif contains the consensus ATT showing that the FISim measure certainly gives greater importance to better conserved positions (for sequence logos see additional file 3: "JASPAR clustering"). We presented the REL family in two clusters, while in Pape et al. [13], this appears together in the same cluster. We obtained the same two TRPs clusters, but added one extra TRP motif (*MYB.ph3*) to one cluster which Pape et al. [13] considered as an outlier. The *MYB.ph3 *motif shares the consensus AAC*G with the motifs in its cluster. The same bZIP cEBP group is provided, although we added six out of the seven ETSs motifs. Here, the common high degree of conservation of the consensus TTCC forces them to belong to the same cluster. We yielded the same two bHLH clusters, but added one bHLH motif (*Arnt-Ahr*), considered as an outlier in Pape et al. [13], as well as the remaining ETS motif to one of the clusters. Pape et al. [13] presented the FORKHEAD and HMG groups in one single cluster in comparison with three homogeneous clusters obtained. Finally, the heterogeneous cluster that we produced comprises one extra FORKHEAD motif *Foxd3 *that does not contain the consensus GTTTA present in the FORKHEAD group.

**Figure 5:**
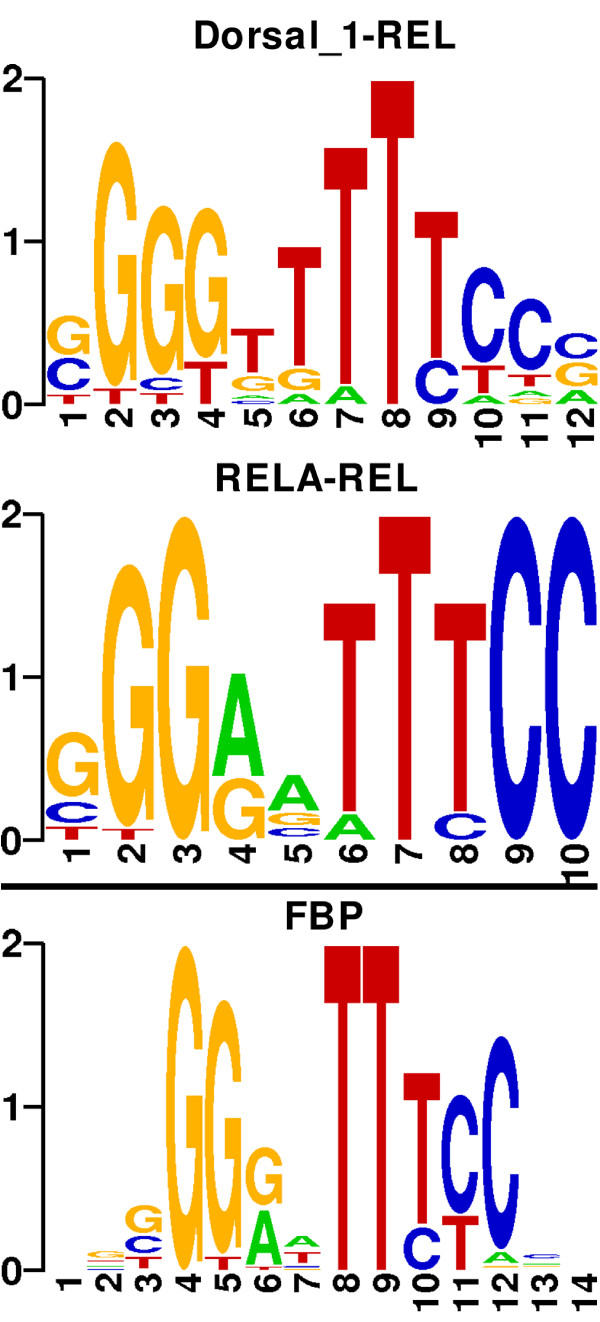
**REL group retrieved by kcmeans**. The FBP is computed from the multiple alignment of the TFs Dorsal_1 and RELA.

In short, we obtained 15 clusters (eleven homogeneous) and found eight outliers (i.e. motifs not clustered), compared to 14 clusters (ten homogeneous) and twelve outliers in Pape et al. [13]. Hence, we found more motifs in the final clustering, reducing the number of non-classified motifs, and maintaining a homogeneous structure. Figure 5 shows the sequence logos of one REL group as well as its corresponding FBP.

### Motif identification in co-regulated genes

As discussed in previous sections, one of the most common applications of a motif similarity measure is its use for comparing putative motifs of co-regulated genes obtained from motif discovery algorithms to those reported in motif databases such as JASPAR or TRANSFAC. In this section we present the results of applying FISim to this workflow with the data studied in [26].

The aim of this study was to classify breast carcinomas based on their gene expression profiling derived from 85 microarray experiments and to correlate tumor characteristics to clinical outcome. The authors classified the tumor samples into two main branches, each of these separated into three subgroups. For this experiment, we selected the "Luminal Subtype A" subgroup, which contains 15 clones (13 genes) clearly involved in pathological processes of breast cancer. This cluster includes genes implicated in transcription, development and differentiation such as *ESR1*, *GATA3*, *LIV1*, and *XBP1 *(see additional file 4: "Motif discovery data" for a whole list of genes).

We applied the motif discovery tool WebMOTIFS [27] to further investigate regulation of the predicted cluster of genes. We used WebMOTIFS to find putative motifs in the promoter regions of these 15 clones, setting the options to default, i.e. selecting AlignACE, MDscan, MEME and Weeder methods [28-31] and no Bayesian information. For each method, we selected the most significant motifs and compared these to the publicly available JASPAR motifs using FISim. Some of the most similar motifs found in JASPAR include ESR1, CREB1, TAL1-TCF3, TP53, NFKB1 and PAX5. For a complete list of motifs, as well as their similarities with JASPAR motifs, see the additional file 4: "Motif discovery data".

As expected, the link between these motifs is the estrogen receptor alpha (*ESR1*) gene. Estrogens play an important role in both female and male reproductive function, as well as in female cancers, and they have multiple effects on the nervous, skeletal, and cardiovascular systems. *ESR1 *is over-expressed in the "Luminal Subtype A" subgroup together with, among others, the *GATA-3*, *LIV-1 *and *XBP1 *genes. Previous studies described how these genes are coordinately expressed with *ESR1 *in breast cancers [32,33]. A wide variety of non-DNA binding molecules, called coactivators, have been identified that are able to enhance ligand-induced activity of steroid receptors, including *ESR1*, through direct or indirect binding to these receptors [34]. Among them, *CREB*-binding protein is critical for ligand-induced, nuclear receptor-mediated transcription activation [35]. In addition, there is evidence that estrogen and progesterone together with *TGF-β *signaling are necessary for maintenance of p53 activity in the mammary epithelium [36], and for an *ESR*-mediated inhibition of the *NFKB *signaling pathway. *NFKB *target genes are significantly elevated in *ESR*-negative versus *ESR*-positive breast tumors, which indicates a potential crosstalk between *NFKB *and *ESR *[37].

## Discussion

We have introduced a new measure of similarity for regulatory motifs called FISim. The uncertainty associated with motif comparison tasks makes fuzzy concepts particularly suitable for handling this kind of data. FISim is based on the fuzzy integral and takes advantage of the fuzzy concepts to overcome some of the known difficulties that arise in measuring motifs tasks. There are three main differences from other approaches: *i) *it considers not only the distance between the PFMs columns, but also the relative importance of each occurrence within each column, *ii) *it enables the inherent uncertainty of the PFMs to be handled, and *iii) *it does not make use of any user-provided parameter.

A simple experiment shows how other measures fail in capturing realistic differences, while FISim provides good results (Figure 2). These results are confirmed on extending the experiment to long datasets (Figure 4). Furthermore, it is noteworthy how the naive euclidean distance [11] inherently appears to assign greater importance to better conserved positions (see Figure 2). This might explain why [12] and [21] found the best performance of their methods when using the euclidean distance to compare the motifs.

As explained above, FISim is based on the fuzzy integral theory. Fuzzy integrals have been proven to be very suitable for information fusion. The combination of the evidence supplied by the information sources (nucleotide frequencies) and the importance of each subset of information sources (nucleotide conservation level) is very interesting in motif recognition tasks. When dealing with long random datasets, we show that FISim provides excellent results in terms of motif recognition, similar to those obtained applying existing methods. This was expected, since the probability of overlapping within random motifs is low, which facilitates the discrimination of the origins of the motifs. Some methods perform poorly when the information contents are low (e.g. ALLR and PCC), however, FISim also provides good results under these circumstances.

This task gets more complicated when motifs are interrelated. In this case, it is noteworthy that the Tomtom algorithm provides very good results for higher information content values. However, FISim provides better results, especially when the information content of the motifs is lower, i.e. when it is more difficult to recognize the motifs. This makes FISim particularly interesting when dealing with real problems. For example, as motif discovery algorithms become more and more powerful, motifs with lower information content will be produced as putative motifs and these will need to be tested.

Another advantage of our method is that it does not require any additional parameter. This makes FISim a more robust and fully automated method, thus avoiding the need to select parameters via expert knowledge or trial-and-error approaches.

We used FISim to investigate the motifs found by popular motif discovery algorithms in a well-known set of co-regulated genes corresponding to the subgroup "Luminal Subtype A" of breast carcinomas.

Comparison of the obtained motifs with those reported in JASPAR suggested that the *ESR1 *gene plays a crucial role in this kind pathology. Furthermore, *ESR1 *interacts with other motifs also present among the most significant motifs obtained. These findings confirm previous studies and show the reliability of FISim in real-life problems.

Our proposed cluster methodology (kcmeans) makes use of FISim and the kernel theory to avoid problems found when applying other classical methods (i.e. definition of a medoid, data order dependence, etc.). The study of the performance of kcmeans in real data shows promising results in terms of accuracy and cluster compactness. Comparison of our results with those from similar experiments shows a better global behavior and a more accurate grouping of the motifs.

## Conclusion

In the present study, we introduce FISim, a new similarity measure for motifs and a novel clustering methodology, based on the fuzzy integral and on kernel technology respectively. Our main objectives were to favour the influence of the better conserved positions of the motifs and to exploit the tolerance for imprecision and uncertainty of fuzzy technology. Our measure takes into account the relative importance of each nucleotide within a given position. We show that FISim outperforms other approaches in motif recognition tasks, and prove how it can be successfully applied to day-to-day research problems. As fuzzy technology is especially suitable for problems that involving imprecise concepts, we are currently working on a fuzzy algorithm that applies the proposed methodology for finding de novo motifs in large sets of DNA sequences.

## Methods

In this section we present our similarity measure and the proposed cluster methodology, and we introduce the concepts used for their definition. A review of the alternative approaches for measuring motif similarities can be found in the additional file 5: "Methodological background".

### Fuzzy Measures

Let *X *= {*x*_1_, *x*_2_...,*x*_*n*_} be a finite set, let *A, B *⊆ *X*, and let ℘(*X*) the power set of *X*. A fuzzy measure, *μ*, is a real valued function *μ *: ℘(*X*) → [0, 1], satisfying the following properties:





The reader should note that the additivity condition of probability theory is relaxed in property 2 to the condition of monotonicity.

For a fuzzy measure *μ*, let *μ*({*x*_*i*_}) = *μ*^*i*^. The mapping *x*_*i *_→ *μ*^*i *^is known as *fuzzy density function*. The fuzzy density of a single element *x*_*i *_∈ *X*, *μ*^*i*^, can be interpreted as the importance of *x*_*i *_in determining the set *X*.

Due to the nature of the definition of a fuzzy measure *μ*, the measure of the union of two disjoints subsets cannot be directly computed from the component measures. In other words, the fuzzy measure value of a subset is not just the sum of the measures of its elements. Therefore, in order to define a fuzzy measure one needs to know not only the individual fuzzy densities of the elements of the measured set, but also the measure for each combination thereof. This information can be supplied by an expert or extracted from the problem definition. However, when dealing with sets of numerous elements this task might become noisy, tedious or even unfeasible. A possible solution for this problem is the use of *λ*-fuzzy measures

#### *λ*-Fuzzy Measures

*λ*-fuzzy measures [19] satisfy the properties of fuzzy measures plus the following additional property: for all *A, B *⊂ *X *and *A *∩ *B *= ∅,

(1)

Furthermore it can be proved that *λ *can be obtained by solving:

(2)

Therefore, applying equation (1) and (2) one will only need to know the individual fuzzy densities of the elements, *μ*^*i*^, (*i *= 1,...,*n*), in order to construct the fuzzy measure.

### Fuzzy Integral

Let *X *= {*x*_1_,...,*x*_*n*_} be a finite set representing a set of *n *information sources. Let *h *: *X *→ [0, 1] represent a function that matches each element of *X *to its evidence. Let's suppose that *h*(*x*_1_) ≥ *h*(*x*_2_) ≥ ⋯ ≥ *h*(*x*_*n*_), if it is not the case for any element, then reorder *X *so that the relation holds, and let *μ *: ℘(*X*) → [0, 1] be a fuzzy measure. Then the fuzzy integral of *h *with respect to fuzzy measure *μ *is

(3)

where *A*_*i *_= {*x*_1_,...,*x*_*i*_}. The reader should note that if *μ *is a *λ*-fuzzy measure, then *μ*(*A*_*i*_) can be obtained applying equation (1).

The fuzzy integral considers the evidence supplied by each element of a given set and the worth of each subset of elements (by means of a fuzzy measure) in its decision making process. This combination of the importance of the sources and the information provided makes the fuzzy integral appropriate for information fusion. Due to its ability to deal with uncertainties associated with the data extracting and processing procedures, it has been widely applied in pattern recognition and classification [19,20].

### FISim

Using PFMs for the representation of the motifs, we propose a novel column-to-column motif similarity measure called FISim (Fuzzy Integral Similarity). FISim is based on the fuzzy integral of the distances of the nucleotide frequencies with respect to the level of conservation of the positions. In our case, the binding preferences of each position (column) are taken as the fuzzy membership degrees to sets of the four DNA nucleotides (A, C, G, T). The reader should note that uniform background distribution is assumed. When dealing with a biased background, PFMs should be modified as stated in [18].

Let  and  be the two columns to be compared. Let  be the set of information sources. To simplify the notation we label the pairs with a single letter so that *X *= {*A, C, G, T*}.

As was stated above, fuzzy integrals need of a function to be integrated (the so-called *h *function). *h *can be defined as , where *i *= {*A, C, G, T*}, i.e. the similarity of the nucleotide *i *in the two columns *C*_1 _and *C*_2_.

In addition, a fuzzy measure is needed to determine the relative importance of the subset of elements being considered. Taking advantage of the properties explained above, we can define a *λ*-fuzzy measure *μ*, constructed from the fuzzy densities of the individual elements *μ*^*i*^. In our case, , where *i *∈ {*A*, *C*, *G*, *T*}, i.e. the maximum level of conservation of the two nucleotides, which favors the importance of better conserved positions. At this point, we can just apply equation (2) to obtain *λ*, and equation (1) to finally obtain the fuzzy measure *μ*. It can be easily proven that *μ *fulfils properties 1 and 2 of the fuzzy measures. Once we have *h *and *μ*, it is a straightforward task to obtain the fuzzy integral applying equation (3).

**Figure 6:**
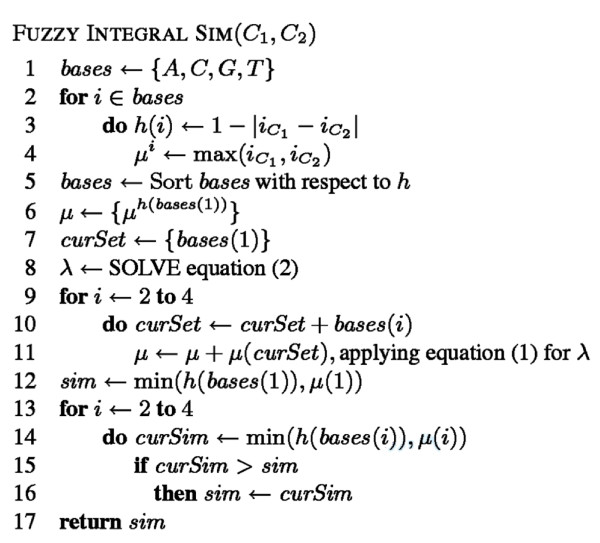
**FISim pseudocode**. This figure shows the pseudocode of the algorithm followed to compute FISim.

Similarity between two PFMs comprising multiple columns needs to be constructed from the aggregation of the column-wise similarities. We proceed by averaging the similarities of the columns considering the best of all possible alignments between the PFMs as well as their reversed complementary sequences. This technique has been shown to work well in previous approaches [9,10]. The algorithm pseudocode can be found in Figure 6. The source code can obtained from . We then provide an example of the computation.

#### FISim example

Let *C*_1 _= (0, 0.9, 0.1, 0), *C*_2 _= (0.1, 0.05, 0.05, 0.8) the columns from the PFMs. *FISim*(*C*_1_, *C*_2_) is obtained as follows: First, we need to compute *h*. Following the formula explained above . Thus, *h*(*A*) = 1 - |0 - 0.1| = 0.9, *h*(*C*) = 1 - |0.9 - 0.05| = 0.15, *h*(*G*) = 1 - |0.1 - 0.05| = 0.95, *h*(*T*) = 1 - |0 - 0.8| = 0.2. Next, *h *is arranged in a decreasing order: {*G*, *A*, *T*, *C*}. From here, the sets *A*_*i *_= {*x*_1_,...,*x*_*i*_} can be obtained: *A*_1 _= {*G*}, *A*_2 _= {*G, A*}, *A*_3 _= {*G, A, T*}, and *A*_4 _= {*G, A, T, C*}.

For the second part of the fuzzy integrals, a fuzzy measure *μ*, is needed. Since we have defined a *λ*-fuzzy measure, we can obtain *μ *from the individual importances *μ*({*x*_*i*_}) = *μ*^*i*^. As we explained above . Hence *μ*^*A *^= 0.1, *μ*^*C *^= 0.9, *μ*^*G *^= 0.1, and *μ*^*T *^= 0.8. Next, we need to obtain the value for the parameter *λ*. This can be done by solving equation (2), for example by applying Newton's method.

In our case *λ *= -0.979. Now, it is easy to compute *μ*(*A*_*i*_) by applying equation (1).

*μ*(*A*_1_) = *μ*({*G*}) = *μ*^*G *^= 0.1, *μ*(*A*_2_) = *μ*({*G, A*}) = *μ*({*G*}) + *μ*({*A*}) + *λμ*({*G*})*μ*({*A*}) = 0.1 + 0.1 - 0.979·0.1·0.1 = 0.190. Similarly, we obtain *μ*(*A*_3_) = 0.841, and *μ*(*A*_4_) = 1.

Now, we are ready to compute the value of the fuzzy integral by solving equation (3). In our case it reduces to *FISim*(*C*_1_*, C*_2_) = max(0.1, 0.190, 0.2, 0.15) = 0.2. Table 2 shows a summary of the computation.

**Table 2:** FISim example

*i*	*h*(*i*)	*μ*^*i*^	*A*_*i*_	*μ*(*A*_*i*_)
*G*	0.95	0.1	*{G}*	**0.1**
*A*	0.9	0.1	*{G, A}*	**0.190**
*T*	**0.2**	0.8	*{G, A, T }*	0.841
*C*	**0.15**	0.9	*{G, A, T, C}*	1

Summary of the computation of the fuzzy integral for the given example (*λ *= -0.979). In bold are the minimum between *h*(*i*) and *μ*(*A*_*i*_). The fuzzy integral value is the maximum value of such minimums, i.e. 0.2.

The reader should note that FISim will assign a high similarity between two columns when their similar values also correspond to well-conserved nucleotides. If a well-conserved position in one column (say 0.9) clearly differs from its corresponding position in the other column (say 0.2), the high value for the importance between these positions (0.9) is ignored. On the contrary, the similarity (0.3) will be the value chosen to proceed with the fuzzy integral computation explained in the previous section.

The reader might ask what are the advantages of FISim over the weighted sum: . Apart from benefits such as the combination of multiple information sources discussed in previous sections, FISim captures much more effectively the concept of similarity in this context, as can be seen in the example.

Computing the weighted sum results: *WA*(*C*_1_, *C*_2_) = 0.9·0.1 + 0.15·0.9 + 0.95·0.1 + 0.2·0.8 = 0.48. This score gives the wrong impression that *C*_1 _and *C*_2 _present medium similarity. On the other hand, the result provided by FISim (0.2) is much more realistic, as the similarity between *C*_1 _and *C*_2 _is expected to be low.

### Kernel C-Means

One of the main applications of motif measures is that they can be incorporated into clustering procedures for grouping related motifs. There exist two previously proposed approaches: application of hierarchical clustering methods [21]; or adaptation of the PAM (Partition Around Medoids) algorithm [13].

Hierarchical methods present problems when dealing with noisy data. They also suffer from a lack of robustness and solutions may be dependent on the data order. Moreover, PAM implementations have the drawbacks that they can converge to local optima and cannot identify clusters that are non-linearly separated in the input space. We propose a novel clustering methodology called kcmeans (kernel c-means) based on the well-known c-means algorithm, kernel methods, and our FISim measure.

The c-means algorithm uses the distances between the objects to group them into clusters. As FISim is a similarity measure, we first need to convert the similarities into distances. If the similarity (*S*) is an inner product, we can compute the distance (*D*) between objects *i *and *j *as *D*_*ij *_= *S*_*ii *_+ *S*_*jj *_- 2 * *S*_*ij*_.

Furthermore, if we want a similarity *S *to be an inner product, we have to force it into a kernel. According to the kernel theory, we can obtain a kernel matrix *S' *preserving the positive eigenvalues and corresponding eigenvectors of *S*. The reader should note that this transformation implies losing some information, however it is expected to be the least significative. The clustering methodology we propose works as follows: we obtain a symmetric matrix of motifs similarities *S *using FISim, we eliminate negative eigenvalues to produce a kernel *S'*, which is an inner product. Finally, we compute the distance matrix *D*_*ij *_= *S*_*ii *_+ *S*_*jj *_- 2 * *S*_*ij *_and then apply c-means to cluster. A review of kernel methods and of the c-means algorithm can be found in the additional file 5: "Methodological background".

## Authors' contributions

FG designed the study, designed and implemented the fuzzy integral similarity, performed the experiments, helped with the analysis of the results and drafted the paper. AB assisted with the design of the study and helped to draft the paper. FJL provided help with the analysis of the results and assisted in drafting the paper. CC implemented the kcmeans clustering methodology, obtained the clustering results and helped to draft the paper. All authors read and approved the final manuscript.

## Supplementary Material

Additional file 1 **JASPAR motifs statistics**. This file contains some statistics obtained from the motifs of the JASPAR database. Click here for file

Additional file 2 **Related motifs experiment**. This file contains the logos of the related motifs experiments as well as the AUC scores. Click here for file

Additional file 3 **JASPAR clustering**. This file contains the description of the clustering obtained by kcmeans and the logos of the motifs for each of the clusters. Click here for file

Additional file 4 **Motif discovery data**. This file contains the supplementary information of the motif identification experiment. Click here for file

Additional file 5 **Methodological background**. This file contains a review of the alternative motif measures, and an introduction to the c-means algorithm and kernel theory. Click here for file
