# Supplementary material for: FISim: A new similarity measure between transcription factor binding sites based on the fuzzy integral
Source: BMC Bioinformatics. 2009 Jul 20;10:224. doi: 10.1186/1471-2105-10-224 (PMC2722654; doi:10.1186/1471-2105-10-224)
Supplement: Additional file 1 — JASPAR motifs statistics. This file contains some statistics obtained from the motifs of the JASPAR database. [file 1471-2105-10-224-S1.pdf]

Fuzzy Integral Similarity for TFBSs. Additional File 1. Jaspar motifs statistics

Jaspar motifs statistics

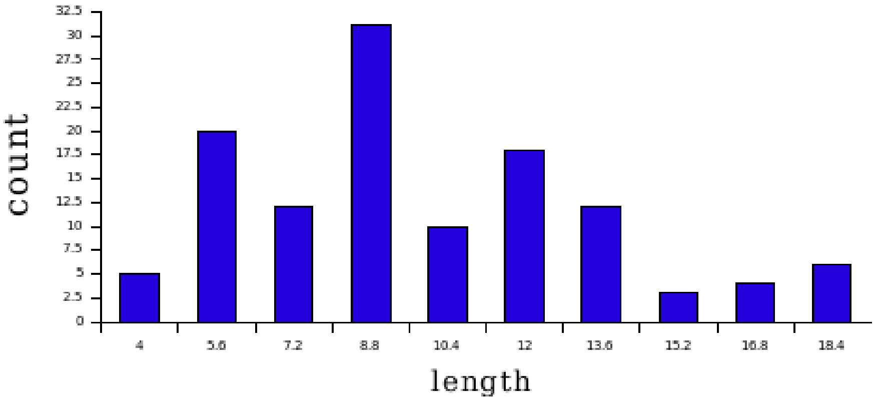

Figure 1: Jaspar motifs length distribution.

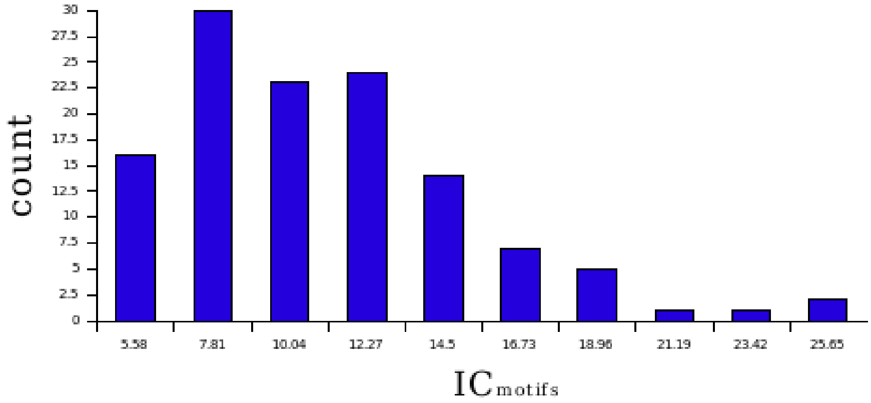

Figure 2: Jaspar motifs IC distribution.

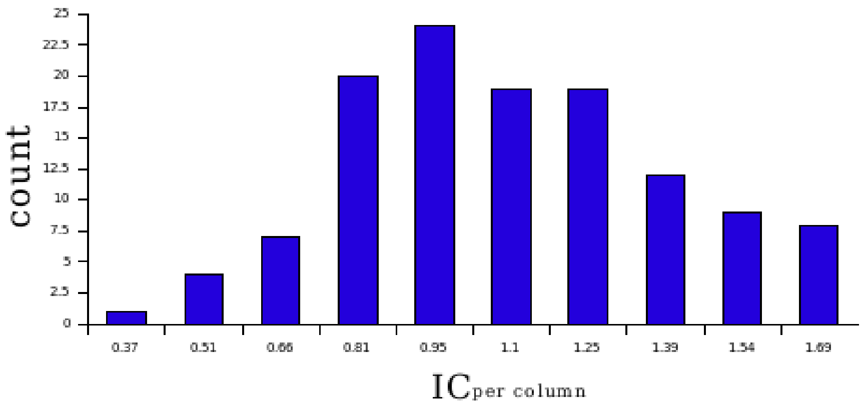

Figure 3: Jaspar motifs IC per column distribution.

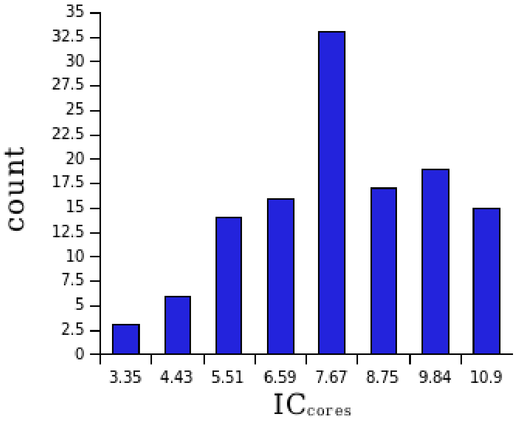

Figure 4: Jaspar cores IC distribution.
