# Supplementary material for: FISim: A new similarity measure between transcription factor binding sites based on the fuzzy integral
Source: BMC Bioinformatics. 2009 Jul 20;10:224. doi: 10.1186/1471-2105-10-224 (PMC2722654; doi:10.1186/1471-2105-10-224)
Supplement: Additional file 2 — Related motifs experiment. This file contains the logos of the related motifs experiments as well as the AUC scores. [file 1471-2105-10-224-S2.pdf]

Fuzzy Integral Similarity for TFBSs. Additional File 2. Related motifs experiment

Related Motifs

In order to facilitate the visual comparison of the non-conserved positions, fraction-based logos are used.

Reference motif

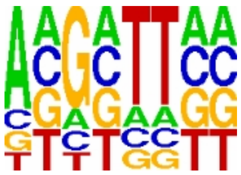

Figure 1: Logo of the motif used as reference in the experiment of related motifs.

Seed motifs

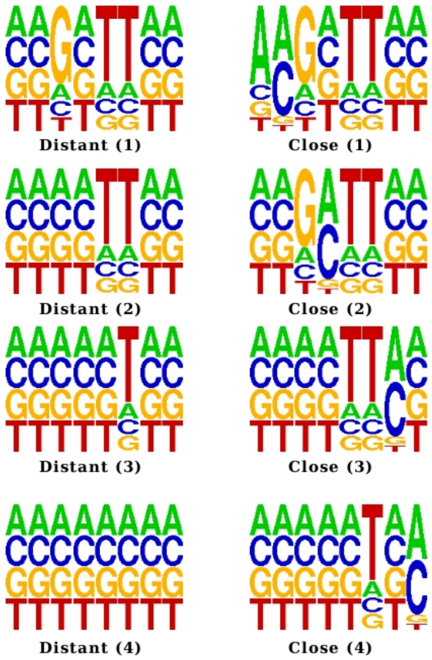

Figure 2: Pairs of seeds of related motifs. The number of the columns not shared with the reference motifs is indicated between brackets

Area Under the Curve Scores

Table 1 shows the area under the curve (AUC) scores of the measures for the related motifs experiment. Note that the more differences between the motifs the more difficult the discrimination task is.

# Differences	FISim	KLD	Chi2	ALLR	Tomtom	Euclidean	Pape
1	0.785	0.328	0.242	0.232	0.894	0.657	0.701
2	0.785	0.350	0.282	0.315	0.795	0.633	0.430
3	0.783	0.353	0.291	0.276	0.697	0.615	0.590
4	0.682	0.202	0.157	0.213	0.573	0.404	0.632

Table 1: Area Under the Curve scores for the related motifs experiment.
