# Supplementary material for: FISim: A new similarity measure between transcription factor binding sites based on the fuzzy integral
Source: BMC Bioinformatics. 2009 Jul 20;10:224. doi: 10.1186/1471-2105-10-224 (PMC2722654; doi:10.1186/1471-2105-10-224)
Supplement: Additional file 3 — JASPAR clustering. This file contains the description of the clustering obtained by kcmeans and the logos of the motifs for each of the clusters. [file 1471-2105-10-224-S3.pdf]

Fuzzy Integral Similarity for TFBSs. Additional File 3. Jaspar Clustering

Jaspar clustering

In this section we describe the results obtained for the application of kcmeans to the Jaspar motifs:

All five TRPs motifs are contained in two homogeneous clusters with two and three motifs. Five of ten BHLHs motifs form one homogeneous cluster. Four of the remaining five BHLHs are grouped together with one ETS motif. All six remaining ETSs motifs form one cluster together with three of four bZIP EBPs motifs. Four of five MADSs motifs are grouped in one homogeneous cluster. The remaining MADS motif is contained in one cluster together with five of six HOMEOs motifs. There exists one heterogeneous cluster formed by the remaining HOMEO motif, one HMG motif and one FORKHEAD motif. Four of the remaining five HMGs motifs are grouped in two homogeneous clusters of two motifs each. Five of the remaining six FORKHEAD motifs form one homogeneous cluster. Seven of eight NUCLEAR motifs form one homogeneous cluster. Finally, all five bZIP CREBs motifs form one homogeneous cluster, and all six RELs motifs form two homogeneous clusters with four and two motifs. Figure 1 shows the clustering result. Logos for the different clusters can be found in the next section. As a summary, ten out of 15 of the obtained clusters are homogeneous, while eight motifs are not clustered and are considered as outliers.

1	IRF1-TRP-CLUSTER	IRF2-TRP-CLUSTER							
2	Dorsal_2-REL	REL-REL	NF-kappaB-REL	NFKB1-REL					
3	FOXF2-FORKHEAD	FOXD1-FORKHEAD	Foxq1-FORKHEAD	FOXI1-FORKHEAD	Foxd3-FORKHEAD				
4	SRY-HMG	Sox5-HMG							
5	Arnt-bHLH	MAX-bHLH-ZIP	MYC-MAX-bHLH-ZIP	USF1-bHLH-ZIP	Mycn-bHLH-ZIP				
6	GAMYB-TRP-CLUSTER	MYB.ph3-TRP-CLUSTER	Myb-TRP-CLUSTER						
7	AGL3-MADS	Agamous-MADS	SQUA-MADS	SRF-MADS					
8	Dorsal_1-REL	RELA-REL							
9	CF1-USP-NUCLEAR	NR2F1-NUCLEAR	PPARG-RXRA-NUCLEAR	PPARG-NUCLEAR	RORA-NUCLEAR	RORA1-NUCLEAR	RXR-VDR-NUCLEAR		
10	SOX9-HMG	Sox17-HMG							
11	CREB1-bZIP	bZIP910-bZIP	bZIP911-bZIP	TCF11-MafG-bZIP					
12	En1-HOMEO	HMG-1-HMG	Foxa2-FORKHEAD						
13	Arnt-Ahr-bHLH	NHLH1-bHLH	Myf-bHLH	TAL1-TCF3-bHLH	SPIB-ETS				
14	Athb-1-HOMEO-ZIP	TCF1-HOMEO	Prrx2-HOMEO	Ubx-HOMEO	Nkx2-5-HOMEO	MEF2A-MADS			
15	NFIL3-bZIP	HLF-bZIP	cEBP-bZIP	E74A-ETS	ELK1-ETS	GABPA-ETS	ELK4-ETS	SPI1-ETS	c-ETS-ETS

Figure 1: Clusters obtained by kcmeans for the Jaspar motifs. The motifs that share the same background color belong to the same Jaspar family.

Logos of the clustering

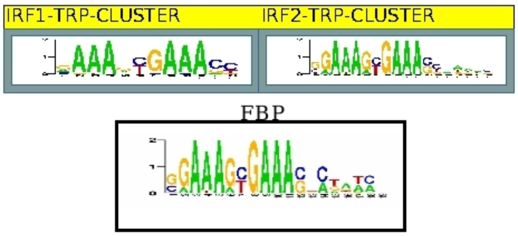

Figure 2: Cluster 1

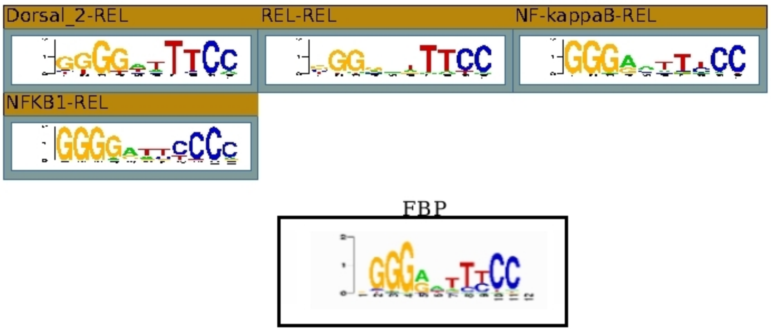

Figure 3: Cluster 2

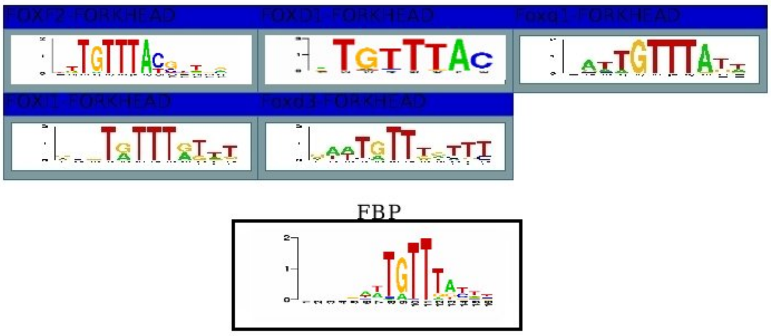

Figure 4: Cluster 3

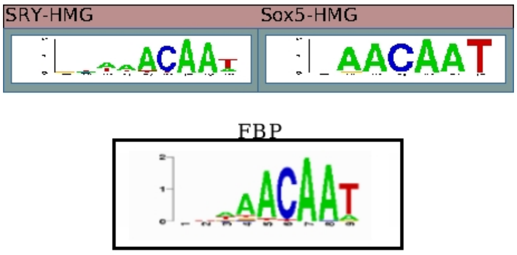

Figure 5: Cluster 4

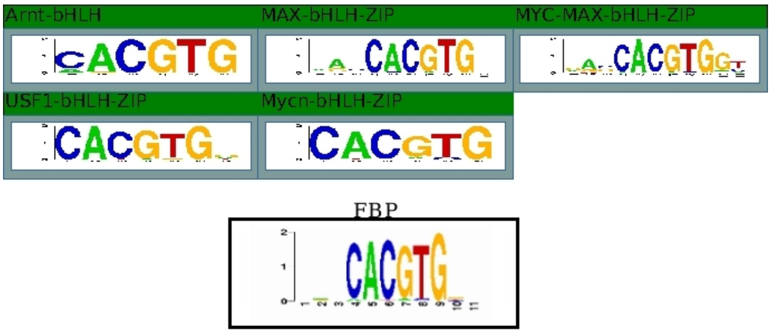

Figure 6: Cluster 5

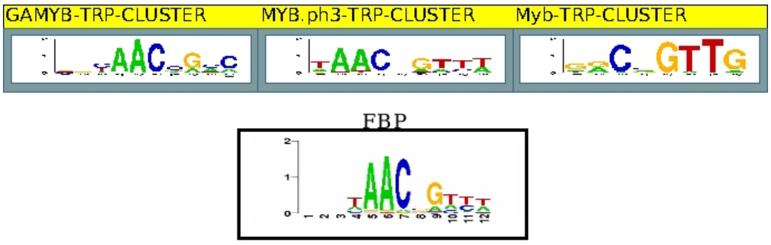

Figure 7: Cluster 6

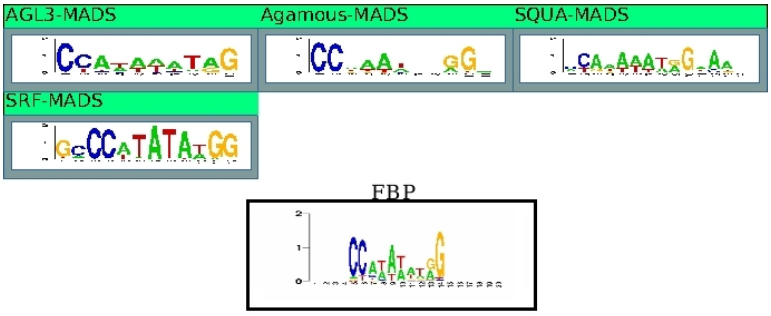

Figure 8: Cluster 7

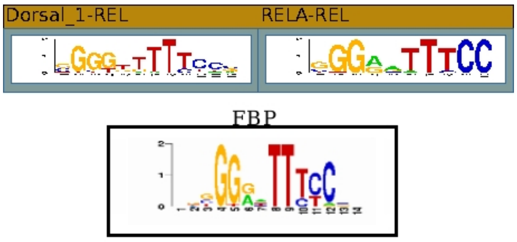

Figure 9: Cluster 8

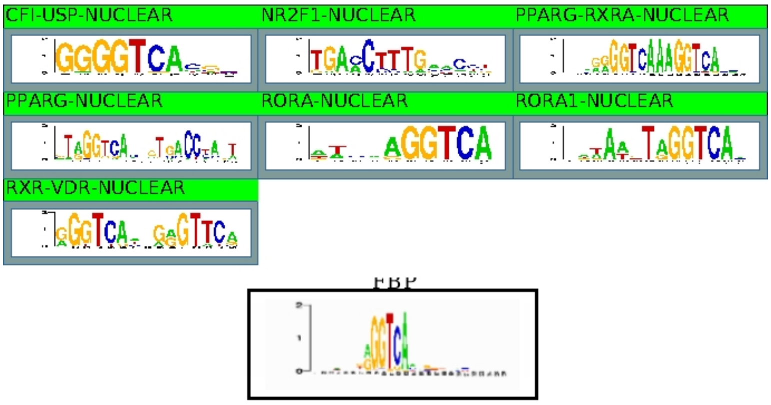

Figure 10: Cluster 9

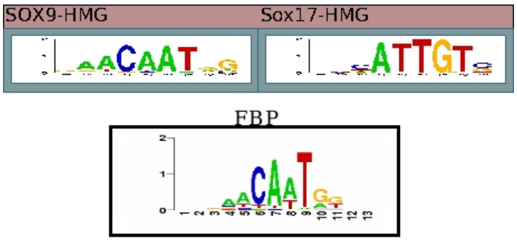

Figure 11: Cluster 10

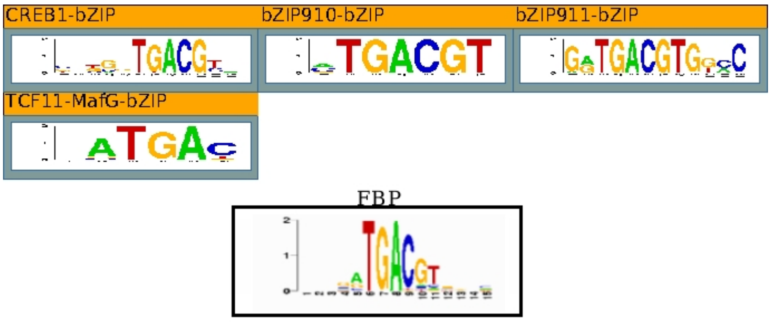

Figure 12: Cluster 11

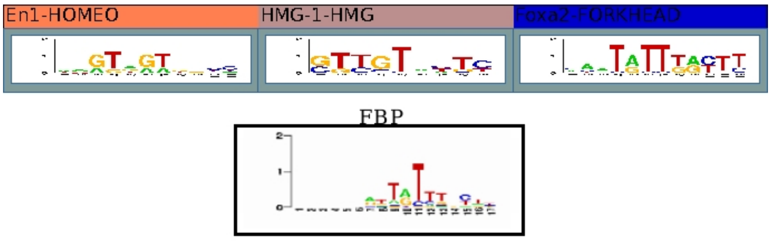

Figure 13: Cluster 12

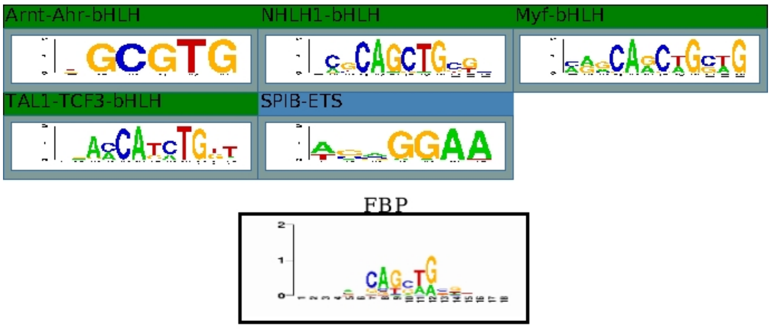

Figure 14: Cluster 13

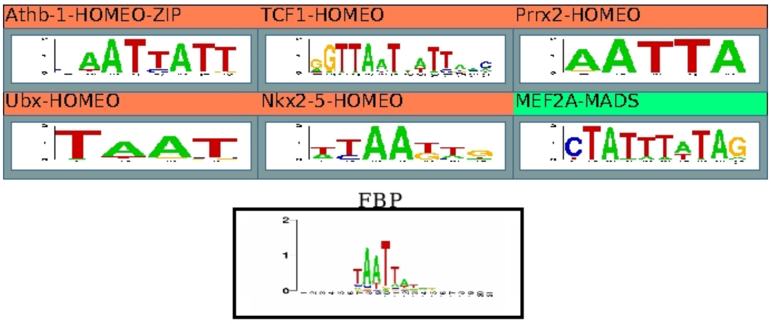

Figure 15: Cluster 14

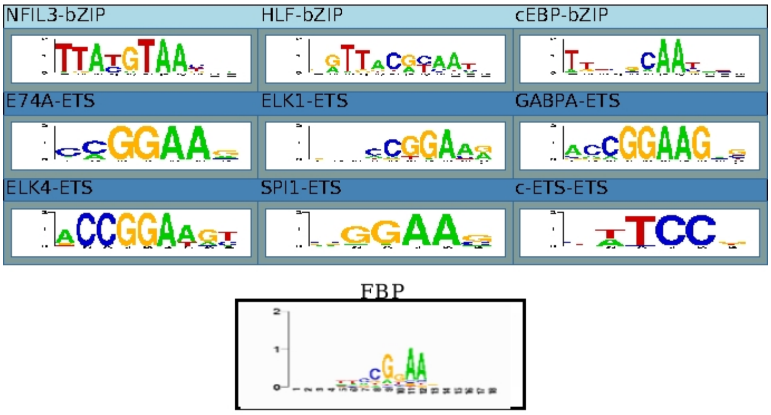

Figure 16: Cluster 15
