# Supplementary material for: FISim: A new similarity measure between transcription factor binding sites based on the fuzzy integral
Source: BMC Bioinformatics. 2009 Jul 20;10:224. doi: 10.1186/1471-2105-10-224 (PMC2722654; doi:10.1186/1471-2105-10-224)
Supplement: Additional file 4 — Motif discovery data. This file contains the supplementary information of the motif identification experiment. [file 1471-2105-10-224-S4.pdf]

Fuzzy Integral Similarity for TFBSs. Additional File 4. Methodological background

1 Alternative approaches

During last years, several measures for comparing motifs have been proposed. In this section we give a brief overview of those that provide better results. The measures are computed for all the possible alignments between the two motifs as well as in their reverse complementary sequences. All but the measure proposed by Pape et al. (1) compound the measure from column-to-column comparisons. In what follows, $C_1 = (A_{C_1}, C_{C_1}, G_{C_1}, T_{C_1})$ and $C_2 = (A_{C_2}, C_{C_2}, G_{C_2}, T_{C_2})$ are the two columns from the PFMs to be compared, b_{C_1} and b_{C_2} ($b \in B, B = \{A, C, G, T\}$) are the probabilities of the base b in C_1, and C_2. $N_{b_{C_1}}$ and $N_{b_{C_2}}$ are the counts of the base b in C_1 and C_2.

Pearson correlation coefficient

Pietrokovski (2) first introduced the Pearson correlation coefficient for comparing motif columns:

$$PCC = \frac{\sum_{b \in B} (b_{C_1} - \overline{C_1})(b_{C_2} - \overline{C_2})}{\sqrt{\sum_{b \in B} (b_{C_1} - \overline{C_1})^2 \sum_{b \in B} (b_{C_2} - \overline{C_2})^2}}.$$

The correlations of all the columns are summarized using the mean.

Average log-likelihood ratio

Wang and Stormo (3) defined the Average log-likelihood ratio (ALLR) statistic to perform motif columns comparisons, which is the sum of two log-likelihood ratios. ALLR is defined as:

$$ALLR = \frac{\sum_{b \in B} N_{b_{C_1}} log\left(\frac{b_{C_2}}{p_b}\right) + \sum_{b \in B} N_{b_{C_2}} log\left(\frac{b_{C_1}}{p_b}\right)}{\sum_{b \in B} (N_{b_{C_1}} + N_{b_{C_2}})},$$

where p_b is the prior for base b. To compare multiples columns, the scores of single columns are summed.

χ^2 test

χ^2 test was proposed by Schones et al. (4) for comparing motifs. This test is computed under the hypothesis that the columns are observations from the

same distribution. The p-value is computed from this χ^2 score with 3 degrees of freedom:

$$\chi^2 = \sum_{j=C_1,C_2} \sum_{b \in B} \frac{(N^o_{jb} - N^e_{jb})^2}{N^e_{jb}},$$

where N^o_{jb} is the observed number of base b at position j, and N^e_{jb} is the expected number of base b at position j (see (4) for more details). The p-value is considered as an additive score.

Kullback-Leibler divergence

Kullback-Leibler divergence has been used to determine similarities between motifs (5). Its symmetric form is:

$$KLD = \frac{1}{2}\left(\sum_{b \in B} b_{C_1} log\left(\frac{b_{C_1}}{b_{C_2}}\right) + \sum_{b \in B} b_{C_2} log\left(\frac{b_{C_2}}{b_{C_1}}\right)\right).$$

Multiple columns are compared averaging column-to-column divergences.

Tomtom

Gupta et al. (6) developed an algorithm (Tomtom) that admits any column-to-column measure to compute the p-values of the match scores for the columns of the query motif aligned with a given target motif. Best results are obtained when using euclidean distance (7). ED is defined as:

$$ED = -\sqrt{\sum_{b \in B} (b_{C_1} - b_{C_2})^2}.$$

In the Tomtom algorithm, a null distribution is approximated in order to obtain a p-value for the sum of the distances for all positions in the motif. The probability of observing a minimum p-value of p^* among a collection of N independent p-values is $1 - (1 - p)^N$. This value is the motif p-value.

Natural measure

Pape et al. (1) defined their measure under the assumption that two motifs should be considered as similar if they yield a high number of overlapping hits on a random sequence, and the number of hits is correlated between both motifs using the asymptotic covariance. Let A and B the motifs to be compared. They compute the score distributions s_A and s_B for the fixed thresholds t_A and t_B. Let $Q^k_{n_A+k}(s_A, s_B)$ be the probability to observe score s_A starting at position j and score s_B starting at position $j + k$ (see (1) for more details). The overlap probability is:

$$\gamma_{A,B}(k) = \sum_{s_A \geq t_A} \sum_{s_B \geq t_B} Q^k_{n_A+k}(s_A, s_B).$$

2 c-means

c-means clustering (8) is a maximization of expectation algorithm that minimize the following cost function:

$$c - means_{cost} = \sum_{i=1}^{c} \frac{\sum_{j=1}^{n} \sum_{k=1}^{n} M_{ji} M_{ki} D_{jk}}{\sum_{l=1}^{n} M_{li}} \tag{1}$$

where c is the number of clusters, n is the number of objects to cluster, D is the pairwise distance matrix, and M is a binary stochastic matrix $M \in \{0,1\}^{n \times k}$ where $M_{ji} = 1$ if object j is in cluster i.

3 Kernel methods.

Given a space X of objects we want to classify, cluster, rank, etc., we can define a function $\phi : X \rightarrow F$, where F is a feature space that eases X classification, clustering, ranking, etc. For example, objects could be more separable in F than in X. Imagine we have a real-valued function $k : X \times X \rightarrow \Re$ and for each $x, y \in X$, $k(x, y)$ tells us how similar x and y are in F. k is called a kernel function and can be defined as the inner product in F: $k(x, y) = \phi(x) \cdot \phi(y)$. In fact, most of the times F is hard or impossible to compute, *e.g.* it could be infinite dimensional. A learning method that uses k to avoid F computation is called a kernel method. More on this topic can be found in (9).

Let us call $P = \{x_1, x_2, ..., x_n\}$ the set of objects to be analyzed. We can construct a kernel matrix $K_{i,j} = k(x_i, x_j)$, $x_i, x_j \in X$. K can be thought as a similarity matrix in F and it is the only way kernel methods access data. For K to be a kernel, it must be semidefinite positive, i.e. all its eigenvalues must be non-negative.

Any learning algorithm that can be formulated in terms of inner products can be interpreted as a kernel method if we replace the inner product with a kernel function. This is known as the kernel trick (9) and allows us to convey kernel ideas to clustering, as can be seen in the original paper.

References

[1] Pape UJ, Rahmann S, Vingron M: **Natural similarity measures between position frequency matrices with an application to clustering**. *Bioinformatics* 2008, **24**:350–357.

[2] Pietrokovski S: **Searching databases of conserved sequence regions by aligning protein multiple-alignments**. *Nucleic Acids Res* 1996, **24**:3836–3845.

[3] Wang T, Stormo GD: **Combining phylogenetic data with co-regulated genes to to identify regulatory motifs**. *Bioinformatics* 2003, **19**:2369–2380.

[4] Schones DE, Sumazin P MQ Zhang: **Similarity of position frequency matrices for transcription factor binding sites**. *Bioinformatics* 2005, **21**:307–313.

[5] Roepcke S, Grossmann S, Rahmann S, Vingron M: **T-Reg Comparator: an analysis tool for the comparison of position weight matrices**. *Nucleic Acids Res* 2005, **33**:438–441.

[6] Gupta S, Stamatoyannopoulos JA, Bailey TL, Noble WS: **Quantifying similarity between motifs**. *Genome Biol* 2007, **8**:R24.

[7] Choi IG, Kwon J, Kim SH: **Local feature frequency profile: A method to measure structural similarity in proteins**. *PNAS* 2004, **101**:3797–2892.

[8] Hartigan J: *Clustering Algorithms.* New York: John Wiley & Sons 1975.

[9] Schölkopf B, Tsuda K, Vert JP: *Kernel Methods in Computational Biology.* Cambridge: The MIT Press 2004.
