# Supplementary material for: FISim: A new similarity measure between transcription factor binding sites based on the fuzzy integral
Source: BMC Bioinformatics. 2009 Jul 20;10:224. doi: 10.1186/1471-2105-10-224 (PMC2722654; doi:10.1186/1471-2105-10-224)
Supplement: Additional file 5 — Methodological background. This file contains a review of the alternative motif measures, and an introduction to the c-means algorithm and kernel theory. [file 1471-2105-10-224-S5.pdf]

Fuzzy Integral Similarity for TFBSs. Additional File 5. Motif discovery data

1 Gene list

Table 1 shows the genes that belong to the cluster termed "Luminal epithelial gene cluster containing ER" in (1).

Gene	GenBank Acc.	Description
GPR160	H50224	G protein-coupled receptor 160
ACADSB	H95792	Acyl-Coenzyme A dehydrogenase, short/branched chain
ESR1	AA291749	Estrogen receptor 1
TFF3	N74131	Trefoil factor 3 (intestinal)
GATA3	R31441	GATA binding protein 3
XBP1	W90128	X-box binding protein 1
FOXA1	T74639	Forkhead box A1
AFF3	H99588	AF4/FMR2 family, member 3
LIV1	H29315	Estrogen-regulated protein LIV-1
NPNT	AA029948	Nephronectin
TUBA1C	N54508	Tubulin, alpha 1c
NAT1	R91802	N-acetyltransferase 1 (arylamine N-acetyltransferase)
MYO6	AA625890	Myosin VI
MYO6	AA030004	Myosin VI

Table 1: List of 15 co-regulated genes

2 Retrieved motifs

In this section we show the most significant motifs obtained from the three motif discovery programs used.

2.1 MDSCAN

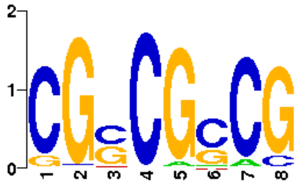

Figure 1: MDSCAN-1

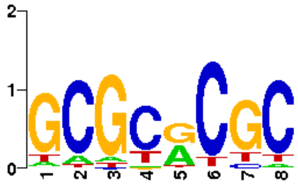

Figure 2: MDSCAN-2

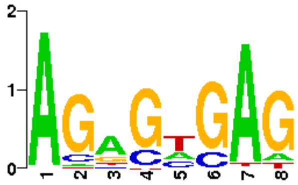

Figure 3: MDSCAN-3

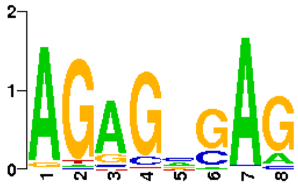

Figure 4: MDSCAN-4

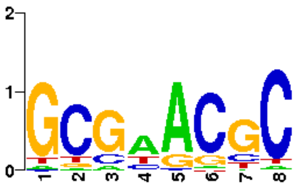

Figure 5: MDSCAN-5

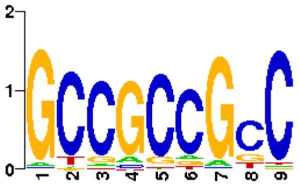

Figure 6: MDSCAN-6

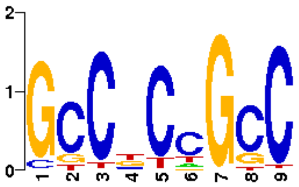

Figure 7: MDSCAN-7

2.2 MEME

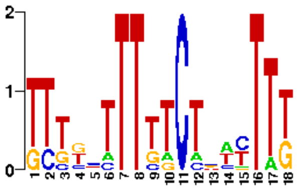

Figure 8: MEME-1

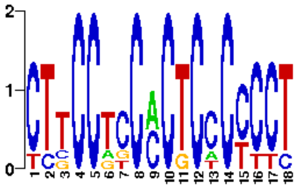

Figure 9: MEME-2

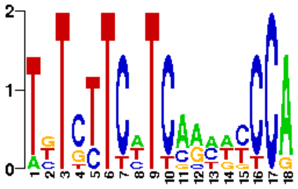

Figure 10: MEME-3

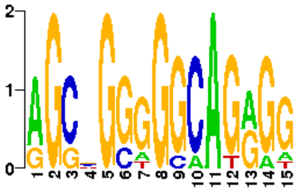

Figure 11: MEME-4

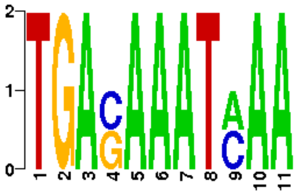

Figure 12: MEME-5

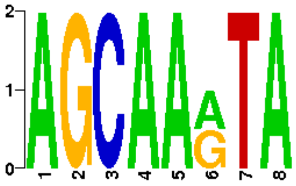

Figure 13: MEME-6

2.3 Weeder

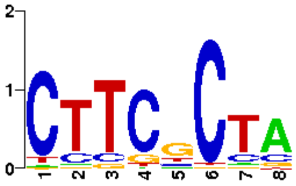

Figure 14: Weeder-1

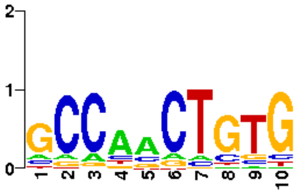

Figure 15: Weeder-2

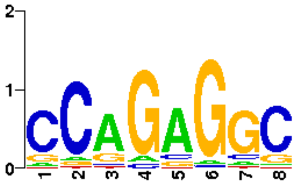

Figure 16: Weeder-3

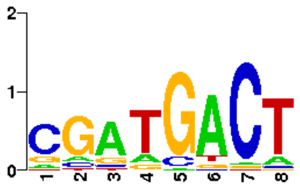

Figure 17: Weeder-4

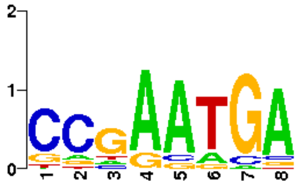

Figure 18: Weeder-5

3 JASPAR matches

In this section we show the best JASPAR matches for each putative motif found by each of the three motif discovery algorithms.

3.1 MDSCAN

Motif	Best JASPAR match	FISim value
MDSCAN-1	CREB1	0.745
MDSCAN-2	ABI4	0.700
MDSCAN-3	ESR1	0.731
MDSCAN-4	TP53	0.732
MDSCAN-5	Pax4	0.704
MDSCAN-6	NFKB1	0.743
MDSCAN-7	TFAP2A	0.701

Table 2: Best JASPAR matches for the MDSCAN algorithm

3.2 MEME

Motif	Best JASPAR match	FISim value
MEME-1	MNB1A	0.790
MEME-2	SPIB	0.793
MEME-3	SPI1	0.808
MEME-4	ZNF42	0.775
MEME-5	GATA2	0.745

Table 3: Best JASPAR matches for the MEME algorithm

3.3 Weeder

Motif	Best JASPAR match	FISim value
Weeder-1	Pax5	0.727
Weeder-2	TAL1-TCF3	0.744
Weeder-3	ESR1	0.682
Weeder-4	TCF11	0.789
Weeder-5	CREB1	0.701

Table 4: Best JASPAR matches for the Weeder algorithm

References

[1] Srlie T, Peroua CM, Tibshirani R, Aas T, Geisler S, Johnsen H, Hastie T, Eisen MB, van de Rijn M, Jeffrey SS, Thorsen T, Quist H, Matesec JC, Brown PO, Botstein D, Lnning PE, , Brresen-Dale A: **Gene expression patterns of breast carcinomas distinguish tumor subclasses with clinical implications**. *PNAS* 2001, **98**(19):10869–10874.
